# Left Atrial Thrombus: a Case Report

**Published:** 2011-11-24

**Authors:** CC Diaconu

**Affiliations:** “Carol Davila” University of Medicine and Pharmacy, Ilfov Clinical Hospital, Internal Medicine Department, Bucharest Romania

**Keywords:** echocardiography, cardiac mass, anticoagulant.

## Abstract

**Rationale:**Echocardiography is essential in establishing the diagnosis in patients with cardiac masses. The differentiation between myxomas and thrombi is sometimes difficult, but is critical in making the right therapeutical decision.

**Objective:** A 70–year–old female presented to the Emergency Department with palpitations, dyspnea and anterior epistaxis. She had a 3 years history of atrial fibrillation and chronic heart failure NYHA class III.

**Method and Result:** Two-dimensional transthoracic echocardiography showed the thickening of the mitral valves with moderate mitral insufficiency and a mobile round mass in the left atrium, heterogeneous, inhomogeneous, 18 mm in size, attached with a narrow stalk to the interatrial septum, reaching mitral annular plane; tricuspid insufficiency with a maximum 30 mmHg gradient, intact interatrial septum, akinesia of two thirds of basal inferior wall, 42% ejection fraction.

**Discussion:** The two–dimensional transesophageal echocardiography confirmed the intraatrial mass. Epistaxis was considered to be due to heart failure and the increased venous pressure. The patient was referred to the cardiovascular surgery clinic, but refused surgery. Anticoagulation with fraxiparine 0,6 ml/day was started and continued for 3 weeks, after cessation of epistaxis by nasal tamponament. Then echocardiography was repeated, with no remnant mass in the left atrium. The conclusion was that the mass must have been a thrombus that has melted away. In this particular case, the left intraatrial thrombus may have been due to the presence of atrial fibrillation.

## Introduction

Echocardiography is essential in establishing the diagnosis in patients with cardiac masses. The differentiation between myxomas and thrombi is sometimes difficult, but is critical in making the right therapeutical decision. When the thrombus moves free in the cardiac cavity the diagnosis is relatively simple [**1,2,3**]. In some cases, the atrial thrombi may have stalk and can be misdiagnosed as myxoma [**1**].

## Methods

A 70–year–old female presented to the Emergency Department with palpitations, dyspnea and anterior epistaxis. She had a 3 years history of atrial fibrillation and chronic heart failure NYHA class III. She was treated with aspirin 100 mg/day. Physical examination revealed an irregular pulse of 148 beats/min, blood pressure of 130/100 mmHg, pansystolic mitral murmur of 2/6 grade, murmur of tricuspid regurgitation of 3/6 grade, lower extremities swelling. The oto-rhino-laryngology exam conclusion was of anterior epistaxis. The 12–lead electrocardiogram revealed atrial fibrillation, inferior ischemia. Her International Normalized Ratio (INR) was of 1,24.

## Results

**Fig. 1:**
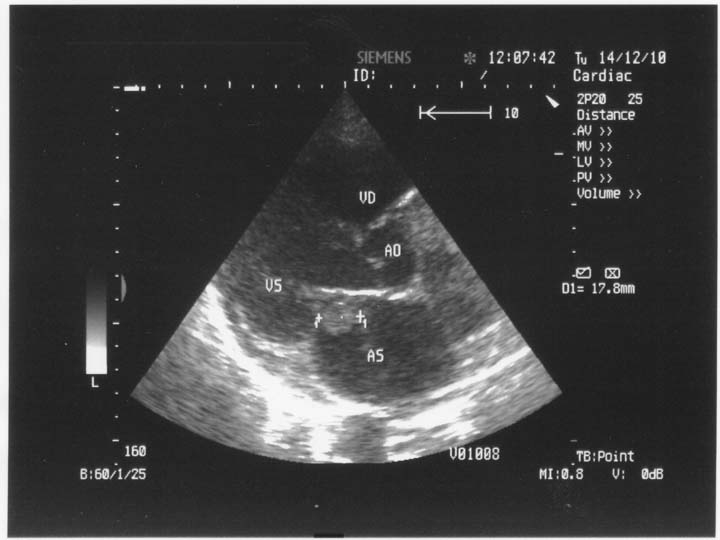
2D Transthoracic echocardiography. Parasternal long axis-view. Left intraatrial thrombus reaching the mitral annular plane. AS=left atrium, VS=left ventricle, VD=right ventricle, Ao=aorta.

**Fig. 2:**
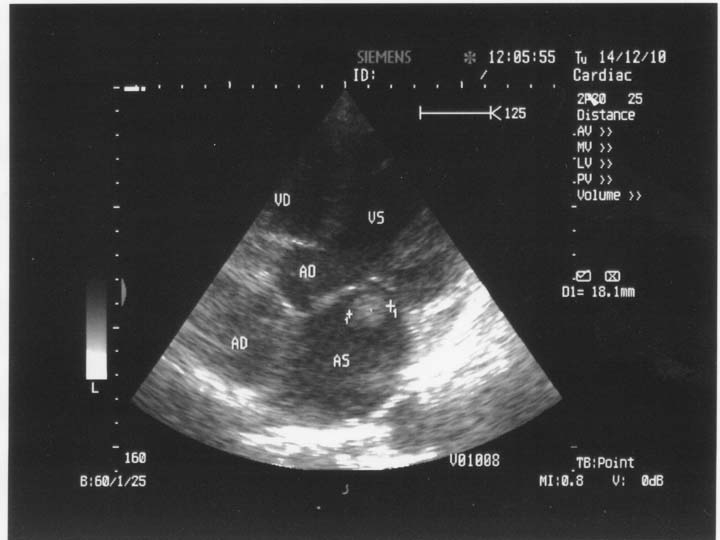
2 D Transthoracic echocardiography. Apical 5 chamber-view. Left intraatrial thrombus between the mitral valves. AS=left atrium, VS=left ventricle, VD=right ventricle, AD=right atrium, Ao=aorta.

The two–dimensional transthoracic echocardiography showed the thickening of the mitral valves with a moderate mitral insufficiency and a mobile round mass in the left atrium, heterogeneous, inhomogeneous, of 18 mm in size, attached with a narrow stalk to the interatrial septum. It showed a tumor–like movement with a cardiac cycle, reaching the mitral annular plane (**Figure 1,Figure 2**).

Also, echocardiography showed tricuspid insufficiency with a maximum gradient of 30 mmHg, intact interatrial septum, akinesia of two thirds of basal inferior wall, ejection fraction of 42%. There was no mass in the left atrial appendage. The two–dimensional transesophageal echocardiography confirmed the presence of the intraatrial mass.

Epistaxis was considered to be due to heart failure and the increased venous pressure.

The patient was referred to the cardiovascular surgery clinic, but she refused surgery. Anticoagulation with fraxiparine of 0,6 ml/day was started and continued for 3 weeks, after cessation of epistaxis by nasal tamponament.

After 3 weeks the echocardiography was repeated, with no remnant mass in the left atrium. The conclusion was that the mass must have been a thrombus that has melted away. In this particular case, the left intraatrial thrombus may have been due to the presence of atrial fibrillation.

## Discussion

Transthoracic and transesophageal echocardiography are the methods of choice for the diagnosis of left intraatrial masses. Transesophageal echocardiography is a superior method in defining the characteristics of these masses. Cardiac myxomas are the most common benign primary tumor of the heart; on echocardiography myxomas appear as mobile masses attached to the endocardial surface by a stalk, usually arising from fossa ovalis. If the stalk is not visible, the diagnosis cannot be made by echocardiography and requires further imaging techniques, like MRI or CT [**1,4**]. In some patients, atrial thrombi may have a stalk and may be mistaken for myxomas, which can lead to unnecessary and potential harmful surgery [**5**]. A left intraatrial mass can be diagnosed as thrombus if it associated with atrial fibrillation, dilated left atrium, mitral or tricuspid stenosis, low ejection fraction, prosthetic mitral or tricuspid valves or spontaneous atrial contrast echoes.

Certain conclusions can be drawn from this case: the differential diagnosis between thrombus and myxoma may be difficult if the left atrial mass has a stalk; when the differential diagnosis is difficult and the possibility of thrombus is higher, the anticoagulation therapy may be a good option, with echocardiographic follow–up.
